# The impact of endoscopic activity on musculoskeletal disorders of high-volume endoscopists in Germany

**DOI:** 10.1038/s41598-022-12400-4

**Published:** 2022-05-20

**Authors:** N. Sturm, J. Leukert, L. Perkhofer, A. Hann, M. Wagner, B. Mayer, T. Seufferlein, J. Mayerle, C. Schulz, A. Meining, E. Kraft, Benjamin M. Walter

**Affiliations:** 1 Department of Internal Medicine I, University Hospital Ulm, Albert-Einstein-Allee 23, 89081 Ulm, Germany; 2 Department of Interdisciplinary Endoscopy, University Hospital Ulm, Ulm, Germany; 3 Department of Internal Medicine II, Interventional and Experimental Endoscopy (InExEn), University Hospital Würzburg, Würzburg, Germany; 4 Department of Orthopaedics and Trauma Surgery, Musculoskeletal University Center Munich (MUM), University Hospital, LMU Munich, Munich, Germany; 5 Medizinische Klinik und Poliklinik II des Klinikums der Universität München Campus Großhadern und Innenstadt, Munich, Germany; 6 Institut für Epidemiologie und Medizinische Biometrie, Universität Ulm, Ulm, Germany

**Keywords:** Gastroenterology, Colonoscopy, Health care, Occupational health

## Abstract

Physical stress is common in GI endoscopists, leading to musculoskeletal disorders. Considering the increasing complexity of interventional GI endoscopy with prolonged examination time, work-related musculoskeletal disorders have come into focus. However, data on work-related health stress in German endoscopists are elusive. The aim of this study was therefore to investigate the prevalence and consequences of work-related musculoskeletal disorders in German endoscopists. A 24-item questionnaire on endoscopy-associated musculoskeletal disorders and standardized pain assessment was developed by an interdisciplinary team of endoscopists and sports medics. The survey was distributed online by the leading German societies for gastroenterology and endoscopy. Overall, 151 German practicing endoscopists took part in the study. Regarding the average number of endoscopic procedures per week, the study collective consisted mainly of high-volume endoscopists. The survey showed that most participants suffered from general musculoskeletal disorders (82.8%) and from work-related musculoskeletal disorders (76.8%). The most affected body parts were the neck, low back, thumb, and shoulder. Temporary absence from work due to symptoms was reported by 9.9% of the respondents. Over 30% of participating endoscopists stated the need for analgesics or physiotherapy due to musculoskeletal disorders. Age, professional experience and work time were identified as relevant risk factors for musculoskeletal health issues. A high number of German endoscopists are affected by musculoskeletal disorders due to specific working postures and repetitive movements with a large impact on personal health. Further interventional studies are mandatory to improve the risk prevention of endoscopic activity.

## Introduction

Musculoskeletal (MSK) health issues have a relevant prevalence in Germany^1^ with a high impact on disability-adjusted life years, years of life lost and years lived with disability, with increasing economic importance in Germany and worldwide^2,3^.

Regarding the development of work-related musculoskeletal disorders, several theoretical models have been proposed to explain and prevent this issue. In their integrated model, Karsh and colleagues have identified environmental, organizational and contextual factors that can lead to work-related MSK health problems^4^. These factors are moderated by individual characteristics such as physical capacity^5^.

Within the context of the high relevance of MSK disorders in work-related health, practicing physicians in gastrointestinal (GI) endoscopy are known to be particularly at risk^6–8^. Several factors have already been identified to predestine endoscopists for MSK disorders. In particular, the combination of repetitive movement and high procedure volume provides the basis of overuse-associated health issues^9–11^.

High pinch force, repetitive hand activities, awkward postures, vibration, and contact stress were found to cause musculoskeletal health disorders among endoscopists by overuse injuries^12^. These overuse injuries can lead to degenerative changes by repeated microtrauma to tendon, ligament, joint and peripheral nerves^13^. As a result, specific health disorders like carpal tunnel syndrome, DeQuervain’s tenosynovitis, and lateral epicondylitis can occure^14,15^.

Due to procedure-specific exposure, the upper extremities (shoulder, wrist, forearm and thumb), the neck and the back are especially at risk for these health issues in GI endoscopy. Furthermore, the impairment of specific body parts differs between different types of endoscopic examinations due to specific body postures and utilized body parts^12^. In general, the endoscope is usually held in the left hand where the left thumb manipulates the navigation panel. While repetitive flexion and extension of the left wrist and the left shoulder navigate the endoscope, the right forearm and shoulder are used to advance the endoscope. Furthermore, the whole spine is involved to focus the monitor which displays the endoscopic image transmission. In consideration of increasingly differentiated new endoscopic interventional and diagnostic approaches, ergonomics in GI endoscopy has not been addressed to the same extent. While other endoscopic disciplines like bronchoscopy are mainly limited to mostly diagnostic interventions^16^, the improvement in GI endoscopic devices and techniques has led to a replacement of conventional surgery in the treatment of early cancerous lesions, e.g. esophageal cancer^17^ and the demand in complex endoscopic interventions has grown. These new interventions do not only extend the overall volume of endoscopic procedures but also entail longer examination times and conditions with potential health hazards comparable to conventional surgery (interventions in general anesthesia, longer examination time and higher technical demands).

For this reason, it can be assumed that the rising complexity of endoscopic interventions along with the inappropriate ergonomic setting could lead to an increase in work-related musculoskeletal health issues in high-volume endoscopists.

Previous studies have investigated the prevalence and consequences of MSK disorders in conventional endoscopists, e.g., in Northern America^6,9,18^, Europe, Portugal^19^, and Asia^20,21^. While the most frequent location of musculoskeletal health issue was comprehensively neck, thumb an back^6,19,21^, the frequency of reported MSK disorders differed from about 50% in US endoscopists^6,9^ to 89.1% in a Korean survey^21^. Despite major differences in sample size and study conduct, these results show that the prevalence of MSK health disorders in GI endoscopy differs between different countries and regions. This might be explained by different practices in conducting endoscopic procedures, overall frequency and proportion of different endoscopic procedures as well as the infrastructure of healthcare provision based on different models of health care systems and frequency of GI disorders.

For example, the indication for screening colonoscopy differs between different countries regarding the recommended age for the first examination and the repetition intervall^22^. The frequency of endoscopic interventions vary through the prevalence of specific GI disorders (e.g., the higher prevalence of gastric cancer in Eastern Asia compared to the North America^23^). Furthermore, even between European countries, working time and the share of endoscopic examination hours in gastroenterology has a large variability^24^.

Considering these features and general work-related differences^25^, a further specific evaluation of population-related musculoskeletal health issues is mandatory.

The aim of this study was to explore the risk factors, impact, and prevalence of musculoskeletal disorders in practicing German endoscopists.

## Materials and methods

An electronic survey was conducted from January 2021 to May 2021 and was addressed to practicing GI endoscopists in Germany. The questionnaire was distributed by the German Society for Gastroenterology (DGVS) (about 6500 members; interventionalists and non-interventional gastroenterologists) and the German Society for Endoscopy and Imaging Methods (DGE-BV) (about 1000 members), which are the leading organizations for gastroenterologists in Germany. To address all German endoscopists, the invitation for study participation was repetitive distributed via e-mail newsletter and the journals associated with the above-named societies (Zeitschrift für Gastroenterologie, endoscopy campus journal). All physicians with regular practice in GI endoscopy and the ability for informed consent were eligible to participate in our survey.

Informed consent was implied by the response to the survey and the aims of our study were explained before the survey could be entered. No financial compensation was given for participation. Answers were initially checked qualitatively by the authors regarding their plausibility (e.g., believable number of performable complex endoscopic interventions and diagnostic examinations per week based on the evaluation of authors with endoscopic experience, exclusion of contradictory data) and the survey could only be transmitted with the confirmation of endoscopic activity. Ethical approval was obtained from the Ethics Committee of the Faculty of Medicine, Ulm University, Ulm, Germany. All methods were performed in accordance with the relevant guidelines and regulations provided by the Ethics Committee of the Faculty of Medicine, Ulm University, Ulm, Germany.

### Survey instrument

A 24-question, self-administered questionnaire was developed by members of the Endoscopic Research Unit (ERU) of Ulm University Hospital, the research group Interventional and Experimental Endoscopy (InExEn) of Würzburg University Hospital and the Department of Orthopaedics and Trauma Surgery, Musculoskeletal University Center Munich.

Survey items were developed by the interdisciplinary study team based on a systematic literature review considering current research on musculoskeletal disorders in practicing endoscopists. The survey was initially distributed, tested and optimized among endoscopists of the Department of Gastroenterology of Ulm University Hospital and the Department of Gastroenterology of Würzburg University Hospital. The survey items were evaluated and adjusted regarding clarity of the question, context and required processing time.

The questionnaire finally evaluated the general characteristics of participating endoscopists, such as sex, age, height, weight, dominant hand, glove size, working place, experience in endoscopic procedures, workload, work breaks and sporting activity. Data on the type and number of endoscopic procedures performed per week were obtained.

Information about musculoskeletal health issues with specific localization and their consequences regarding required medical treatment, absence from work and changes in endoscopic practice was additionally collected.

In case of musculoskeletal health issues, a validated region-specific questionnaire was added to the basic 24-item questionnaire for each pain localization. In general, specific questions contained pain intensity and impairment of activities of daily life. All region-specific questionnaires were selected by an interdisciplinary team of sport orthopaedics and endoscopists and have been validated in previous studies regarding their psychometric properties (reliability, validity, responsiveness).

For health issues concerning arm, shoulder and hand, the validated Quick-DASH (Disability of the Arm, Shoulder, Hand) Index by Germann et al. was used^26–28^. A high internal consistency (Cronbach’s alpha 0.91) was assessed for the German version by Angst et al. (*n* = 320 patients)^29^ and a good validity and responsiveness regarding work-related musculoskeletal health issues has been demonstrated by Stover et al. al for the original version (*n* = 559 patients with work-related upper extremity musculoskeletal disorders)^30^.

Musculoskeletal disorder of the neck was assessed by the Neck Disability Index by Cramer et al.^31–33^. Regarding the reliability, a Cronbach’s alpha of 0.81 for internal consistency as well as a good convergent validity (significant correlation with pain intensity, pain on movement, health-related quality of life, and range of motion) and responsiveness could be demonstrated by Cramer et al. (*n* = 51 neck pain patients) for the German version^31^.

The Roland and Morris Disability Questionnaire was used to obtain information about health complaints considering the lower back^34–36^. Wiesinger et al. have tested the German version of the Roland and Morris Disability Questionnaire on 125 patients with lower back pain leading to a good intern consistency (Cronbach’s alpha 0.81) and validity for pain intensity and lateral and forward bending (*p* = 0.0001)^35^. A good responsiveness for the 24-items version has been demonstrated by Macedo et al. (*n* = 1069 lower back pain patients)^37^.

The Western Ontario and McMaster Universities Osteoarthritis Index (WOMAC) was integrated into the survey to evaluate health issues concerning the knee and hip^38–40^.

For the German version, a good reliability regarding the intern consistency (Cronbach’s alpha 0.81–0.96 for all scales) was demonstrated by Stucki et al. (*n* = 51). In a systematic literature research, McConnell et al. have stated a sufficient validity and responsiveness^41^.

Overall, a maximum of 93 items depending on reported musculoskeletal health issues were used.

### Survey administration

The electronic survey was distributed by the German Society of Gastroenterology (Deutsche Gesellschaft für Gastroenterologie, Verdauungs- und Stoffwechselkrankheiten) and the German Society for Endoscopy and Imaging Methods (Deutsche Gesellschaft für Endoskopie und Bildgebende Verfahren) via e-mail to all members of society and receivers of the online newsletter, as well as on the respective websites and in the associated journals of both societies (Zeitschrift für Gastroenterologie, endoscopy campus). The questionnaire was transferred into an online survey using the SurveyMonkey (https://www.surveymonkey.de/) survey tool (Momentive Inc., San Mateo, California, USA)^42^. Furthermore, the SurveyMonkey survey tool was used for data collection and extraction. No participant-related personal data were obtained within the study. All the answers remained anonymous.

## Statistical analysis

All data were arranged, processed and analysed with SPSS Statistics 21 (IBM, USA) after collection and extraction by SurveyMonkey tool. Categorical variables were described through absolute and relative frequencies, and continuous variables were described through the mean, standard deviation, minimum and maximum.

To identify risk factors for musculoskeletal health issues in German endoscopists, binary logistic regression was used for each variable. The likelihood of developing musculoskeletal health issues (binary dependent variable; 0 = no musculoskeletal health issues, 1 = musculoskeletal health issue) was investigated for categorially and metrically scaled variables with expected impact. The direction of the influence (towards risk reduction or increase for MSK disorders) was obtained by the regression coefficient (positive or negative). Odds ratio was used to interpret the probability of suffering from work-related musculoskeletal health issues depending on different independent variables (age, sex, professional experience, working time in endoscopy, height, weight, BMI, physical exercise during leisure time, glove size, breaks from work, place of work, number of endoscopic examinations per week by total number and specific procedure). Odds ratio > 1 indicated a higher probability of suffering from work-related health issues due to the corresponding independent variable while an Odds ratio < 1 indicated an inverse relationship. Statistical significance of the investigated relations was evaluated by considering the *p*-value. A *p*-value < 0.05 indicated statistical significance.

The impact of stated musculoskeletal health issues on daily life was measured and interpreted by the specific questionnaires mentioned above according to their validated instructions^26,31,34,38^.

## Results

A total of 151 German endoscopists responded to this online survey (5% of all DGVS members, 15% of all DGE-BV members; double membership possible). All respondents could be included in the data analysis after qualitative control regarding the plausibility of the given answers.

Characteristics are summarized in Tables 1 and 2.

**Table 1:** Demographic data of participating endoscopists.

Characteristic	Frequency (*n* = 151)	Percentage (%)
**Sex**		
Male	110	72.8
Female	37	24.5
Missing data	4	2.6
**Dominant hand**		
Right	128	84.8
Left	9	6.0
Both	7	4.6
Missing data	7	4.6
**Glove size**		
S	22	14.6
M	36	23.8
L	66	43.7
XL	21	13.9
Missing data	6	4.0
**Level of training**		
Specialist	133	88.1
Residency	13	8.6
Missing data	5	3.3
**Place of work**		
Specialist practice/day clinic	42	27.8
Community hospital	80	53.0
University hospital	23	15.2
Missing data	6	4.0
**Breaks**		
None	34	22.5
Occasionally	82	54.4
Regular	29	20.0
Missing data	6	4.0
**Physical exercise**		
None	29	19.2
1–2 times per week	78	51.7
3–4 times per week	30	19.9
Daily	8	5.3
Missing data	6	4.0

**Table 2:** Demographic data of participating endoscopists (*n* = 151).

Characteristic	Mean ± SD	Range
Age (years)	49.4 ± 10.4	28–80
Height (cm)	178.0 ± 9.1	151–200
BMI (kg/m^2^)	24.8 ± 3.1	17.8–42.2
Weight (kg)	78.9 ± 12.8	49–122
Professional experience (years)	21.0 ± 10.1	5–46
Working time in endoscopy (hours/day)	6.2 ± 2.1	1–12
Number of endoscopic examinations per week (total)	86.4 ± 38.3	14–198
**EGD**	32.1 ± 17.1	5–105
Diagnostic	23.6 ± 11.6	5–70
Therapeutic	8.6 ± 7.6	0–35
**Intestinoscopy**	2.4 ± 6.0	0–35
**Colonoscopy**	32.5 ± 18.9	0–95
Diagnostic	21.0 ± 13.4	0–70
Therapeutic	11.4 ± 7.6	0–35
**EUS**	5.8 ± 6.4	0–30
Diagnostic	4.3 ± 4.8	0–25
Therapeutic	1.5 ± 2.5	0–17
**ERCP**	4.1 ± 5.0	0–27
Cholangioscopy	0.5 ± 1.2	0–10
**Special Intervention**	4.7 ± 5.7	0–29
EMR	4.7 ± 5.7	0–31
ESD	0.4 ± 1.3	0–10
POEM	0.1 ± 0.3	0–2
FTR	0.4 ± 0.7	0–4
**Other**	4.6 ± 10.2	0–66

*EGD* Oesophagogastroduodenoscopy, *ERCP* endoscopic retrograde cholangiopancreatography, *EUS* endoscopic ultrasound, *ESD* endoscopic submucosal dissection, *EMR* endoscopic mucosal resection, *POEM* peroral endoscopic myotomy, *FTR* full-thickness resection, *BMI* body mass index.

The respondents were predominantly male (*n* = 110; 72.8%), with an average age of 49.9 years and a mean BMI of 24.8 kg/m^2^. The majority of participating endoscopists were endoscopic specialists (*n* = 133; 88.1%) working in a community hospital (*n* = 80; 53.0%) or a specialist outpatient clinic (n = 42; 27.8%). The mean professional experience of the contributors was 21.0 years, with an average active working time of 6.2 h per day in endoscopic facilities.

**Figure 1:**
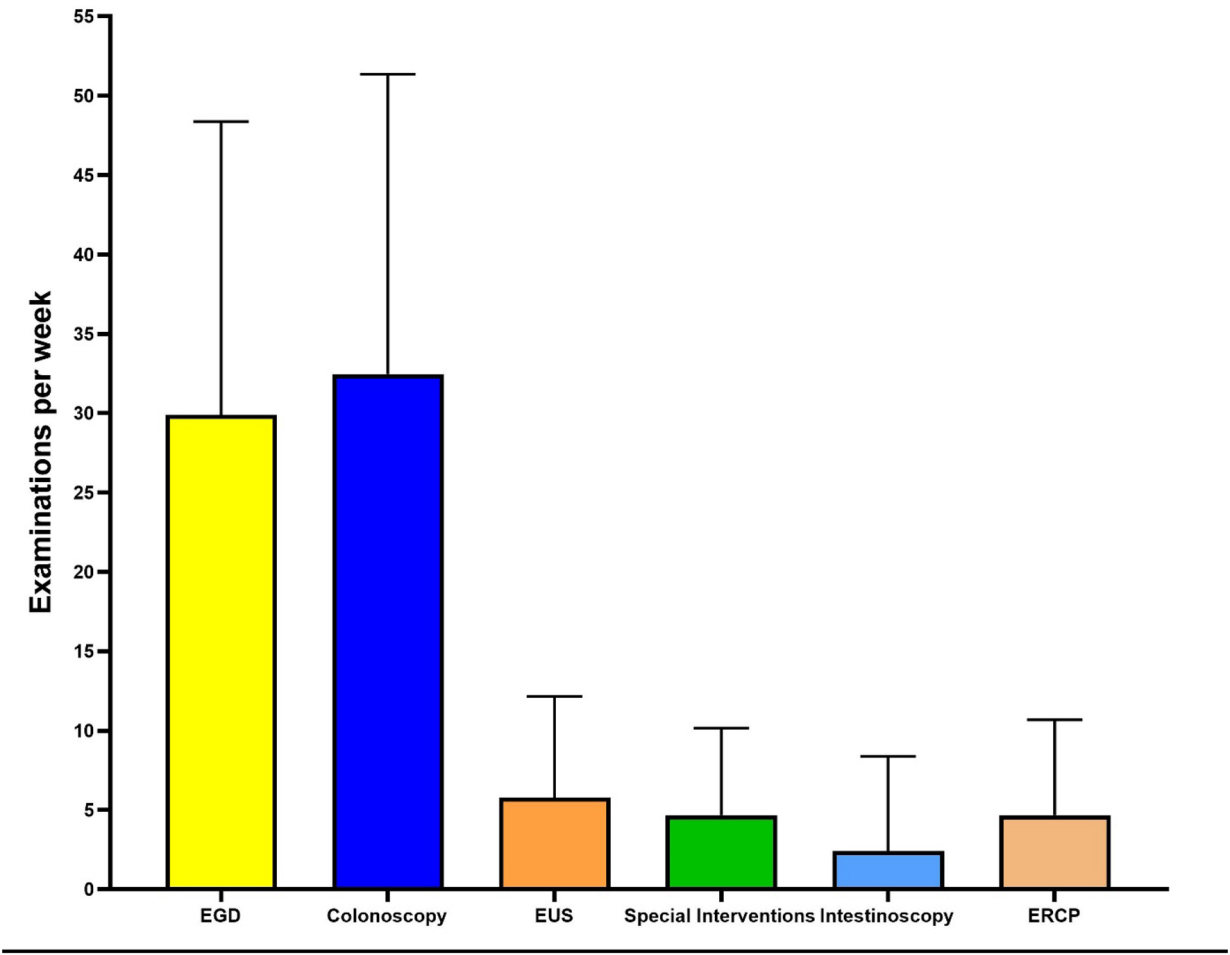
Number of endoscopic procedures per week (medium and maximum) of German endoscopists by examination category. Special interventions included ESD, EMR, POEM and FTR. *EGD* Oesophagogastroduodenoscopy, *ERCP* endoscopic retrograde holangiopancreatography, *EUS* endoscopic ultrasound, *ESD* endoscopic submucosal dissection, *EMR* endoscopic mucosal resection, *POEM* peroral endoscopic myotomy, *FTR* full-thickness resection.

The average number of examinations per week was 86.4 per endoscopist, which shows that the study collective mainly entailed high-volume GI endoscopists. Details of the performed endoscopic procedures are shown in Table 2 and Fig. 1.

Regarding potential protective factors, working breaks were mostly taken occasionally (*n* = 82; 54.2%) or absent (*n* = 34; 22.5%). Only twenty-nine endoscopists took regular breaks from work (20.0%). During leisure time, participants are active in sports mostly once or twice per week (*n* = 78; 51.7%). Twenty-nine respondents stated that they were doing no sports at all (19.2%).

**Figure 2:**
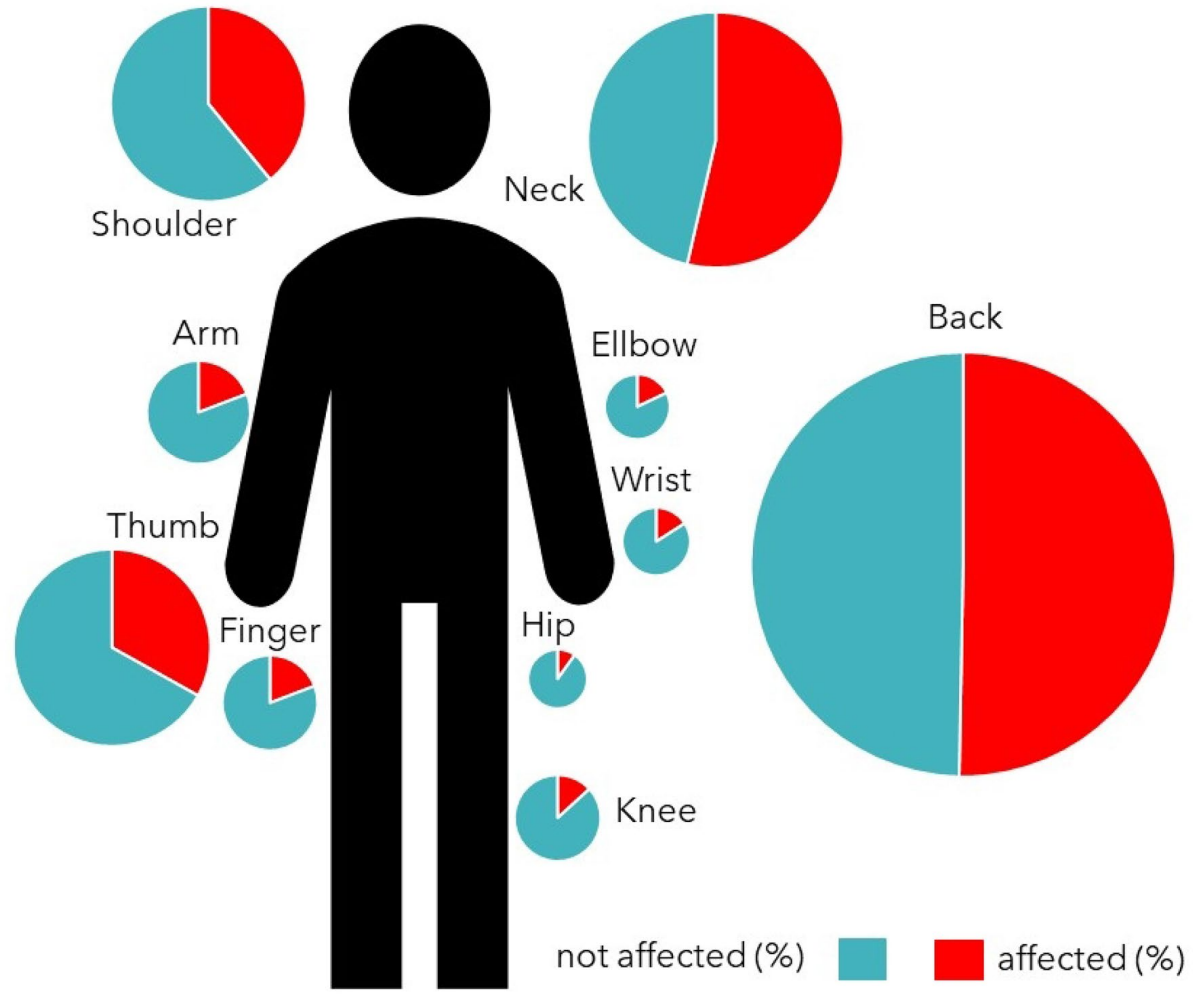
Musculoskeletal health disorder in German endoscopists by localization (%).

In total, 125 respondents (82.8%) dealt with musculoskeletal health issues, and 116 (76.8%) stated that these problems were associated with their endoscopic activity. The most affected body parts were the neck (*n* = 81; 53.6%), back (*n* = 76; 50.3%), shoulder (*n* = 59; 39.1%) and thumb (*n* = 50; 33.1%). The distribution among different affected body parts is summarized in Fig. 2.

The quality of impairment and pain intensity of specific body parts were evaluated as mentioned above. The evaluation of MSK neck disorder showed a strong intensity with a mean neck disability index score of 34.8% (SD ± 8.1), while problems of the upper and lower extremities and the lower back were mostly less severe. Further details about musculoskeletal problems of the upper extremities (Quick-DASH-Score), the lower extremities (WOMAC-Score), the back (RMDQ-Score) and the neck (Neck Disability Index Score) are shown in Tables S1–S4.

Regarding the impact of work-related MSK disorder on personal health and economic consequences, data reveal that every tenth participating endoscopist was already absent from work due to musculoskeletal health issues (*n* = 15; 9.9%). More than every third respondent (*n* = 54; 35.8%) stated that there was a need for medical therapy. 39 (25.8%) endoscopists had a history of analgesic use, and 39 (25.8%) participants required physiotherapy. In five (3.3%) cases, musculoskeletal disorder even led to surgical treatment.

**Figure 3:**
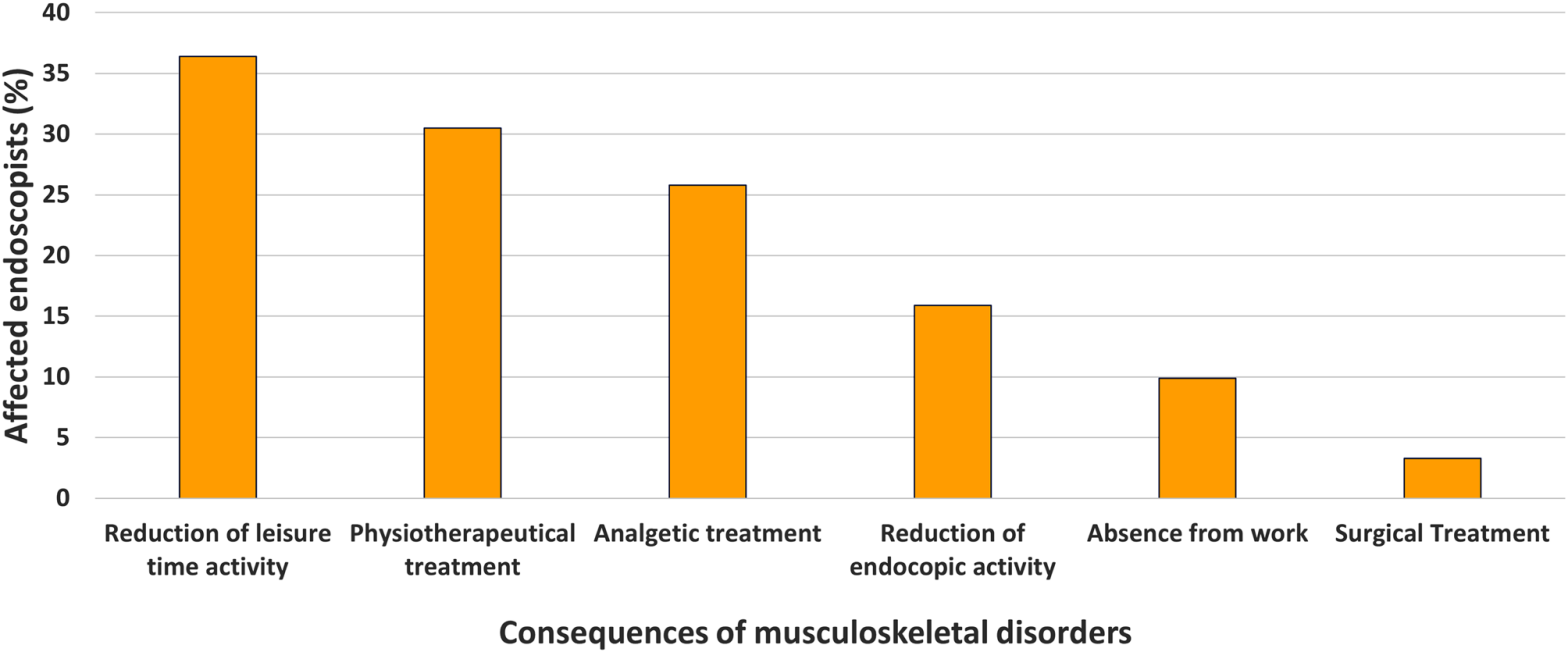
Consequences of musculoskeletal health disorder in German endoscopists (%).

Consequently, 24 (15.9%) participating physicians had to reduce the number of endoscopic procedures, and 55 (36.4%) respondents stated an impairment of leisure time activity due to work-related MSK disorder. An overview of musculoskeletal health issues and consequences is shown in Table 3 and Fig. 3.

**Table 3:** Musculoskeletal health issues relating to activity in endoscopy.

Characteristic	Frequency (*n* = 151)	Percentage (%)
**Musculoskeletal problems (total)**	125	82.8
Musculoskeletal problems associated with endoscopic activity	116	76.8
**Location**		
Neck	81	53.6
Shoulder	59	39.1
Arm	29	19.2
Elbow	27	17.9
Wrist	24	15.9
Thumb	50	33.1
Finger	29	19.2
Back	76	50.3
Hip	15	9.9
Knee	20	13.2
Other	6	4.0
**Absence from work due to musculoskeletal problems**	15	9.9
**Need for medical therapy**	54	35.8
Medication (Analgesics)	39	25.8
Physiotherapy	46	30.5
Surgery	5	3.3
Other	7	4.6
**Reduction in performed endoscopic procedures necessary**	24	15.9
**Reduction of leisure time activity due to endoscopy-associated musculoskeletal problems**	55	36.4

In response to endoscopy-associated health hazards, participants stated different methods of protective interventions, which are summarized in Table 4. These interventions included height adjustment of the examination bed (*n* = 42; 27.8%) and endoscopy video monitoring (*n* = 36; 23.8%), changes in endoscopic techniques (*n* = 55; 36.4%) and the implementation of regular breaks from work (*n* = 10; 6.6%). Other improvements in personal health included a reduction in examination volume (*n* = 5; 3.3%), the use of orthopedic aids (*n* = 10; 6.6%) and specific physical exercise during leisure time (*n* = 17; 11.3%).

**Table 4:** Changes at work in response to endoscopy-associated musculoskeletal problems.

Intervention	Frequency (*n* = 151)	Percentage (%)
Height adjustment of the examination bed	42	27.8
Height adjustment of the video monitor	36	23.8
Changes in endoscopic technique	55	36.4
Regular work breaks	10	6.6
Reduction in the number of daily examinations	5	3.3
Orthopedic aids	10	6.6
Physical exercise	17	11.3

A total of 80.1% (*n* = 121) of all respondents stated the mandatory requirement for ergonomic improvement of their work environment, and 78.1% (*n* = 118) confirmed the demand for specific preventive physiotherapeutic exercise and support.

To evaluate predictive factors for work-related musculoskeletal health issues, binary logistic regression was performed with demographic and work-related data. Consequently, the age of the participants could be identified as an independent risk factor with a mild effect on work-related health hazards (OR 1.05, CI 1.00–1.09; *p* = 0.04). Furthermore, the working time in endoscopy (hours/day) led to a mild risk increase for musculoskeletal health problems with a barely nonsignificant effect (OR 1.20, CI 1.00–1.37, *p* = 0.05) as well as professional experience in years (OR 1.04, CI 1.00–1-09; *p* = 0.06). Moreover, all other obtained demographic and work-related data showed no predictive effect on work-related musculoskeletal health issues (summarized in Table 5).

**Table 5:** Logistic regression model of predictors of endoscopy-associated musculoskeletal problems.

Variable	Odds Ratio	95% CI	Significance
Age (years)	1.05	1.00–1.09	**0.04**
Sex	0.85	0.31–2.30	0.75
Professional experience (years)	1.04	1.00–1.09	0.06
Working time in endoscopy (hours/day)	1.20	1.00–1.37	**0.05**
Height (cm)	1.02	0.97–1.07	0.45
Weight (kg)	1.00	0.97–1.04	0.72
BMI (kg/m^2^)	1.00	0.87–1.14	0.99
Physical exercise	1.49	0.87–2.54	0.14
Glove size	0.86	0.55–1.36	0.52
Breaks	1.34	0.71–2.54	0.37
**Place of work**			
Specialist practice/day clinic	0.63	0.19–2.11	0.45
Community hospital	0.53	0.18–1.62	0.27
University hospital	1.59	0.47–5.37	0.45
Number of endoscopic examinations per week (total)	1.00	0.99–1.02	0.69
**EGD**	1.01	0.99–1.03	0.39
Diagnostic	1.01	0.98–1.05	0.43
Therapeutic	1.02	0.97–1.08	0.50
Intestinoscopy	1.04	0.98–1.10	0.23
**Colonoscopy**	0.99	0.97–1.01	0.42
Diagnostic	0.99	0.96–1.02	0.47
Therapeutic	0.98	0.92–1.04	0.45
**EUS**	0.99	0.93–1.06	0.83
Diagnostic	0.98	0.89–1.07	0.60
Therapeutic	1.04	0.89–1.22	0.61
**ERCP**	1.01	0.93–1.10	0.80
Cholangioscopy	1.13	0.83–1.54	0.43
**Special Intervention**	0.99	0.91–1.08	0.84
EMR	0.99	0.92–1.08	0.89
ESD	1.22	0.91–1.62	0.18
POEM	0.57	0.08–4.13	0.58
FTR	0.53	0.21–1.33	0.17
Other	1.02	0.98–1.06	0.28

*EGD* Oesophagogastroduodenoscopy, *ERCP* endoscopic retrograde cholangiopancreatography, *EUS* endoscopic ultrasound, *ESD* endoscopic submucosal dissection, *EMR* endoscopic mucosal resection, *POEM* peroral endoscopic myotomy, *FTR* full-thickness resection, *BMI* body mass index.

## Discussion

The present study evaluated the prevalence of musculoskeletal health disorders and possible risk factors in German GI endoscopists. Regarding the average number of endoscopic examinations per week, it can be stated that the participants were mainly high-volume endoscopists. It was demonstrated that most of the participating physicians had already suffered from musculoskeletal health issues (*n* = 125; 82.8%), which was mostly attributed to endoscopic activity (*n* = 116; 76.8%). The most affected body areas were the shoulders, thumb, neck and back due to endoscopy-associated overuse. These results of commonly affected body parts by overuse injury in GI endoscopy correspond to the findings of previous studies (see below). While the upper extremities and the thumb are mainly strained by handling the endoscope, neck and back are affected by awkward positions during endoscopic procedures and by focusing the video monitor. Consequently, our results show that after the occurrence of MSK health disorders, up to over one third of survey participants changed their endoscopic techniques and improved the ergonomic features of their workplace. These results suggest that before developing MSK health issues, many endoscopists adopted endoscopic techniques and a workplace design with major deficits in ergonomics that ultimately led to MSK health disorders. Especially an inadequate high adjustment of the video monitor and the examination bed was changes after the occurrence of MSK health disorder. Furthermore, reduction of examination time and the implementation of regular work breaks and physical activity during leisure time were introduced by the affected endoscopists.

To investigate the mechanisms of specific MSK health disorders in practicing GI endoscopists, further observational and interventional studies are mandatory to identify mechanisms of injury and opportunities of prevention. Regarding the analysis of pain intensity by specific pain assessments, disorders of the neck have the strongest qualitative manifestation which suggest that endoscopists might predominantly benefit from ergonomic training in adequate body posture and adjustment of the video monitor.

The high impact of musculoskeletal health issues in German endoscopists was demonstrated by the fact that almost every 10th endoscopist had to stay absent from work due to musculoskeletal health issues. Furthermore, more than every third endoscopist needed medical treatment due to the investigated problems, together with a reduction in daily working volume and leisure time activity. These data on work-related musculoskeletal health issues in German endoscopists are in line with a previous European-wide survey^19^.

Considering potential risk factors, it has been demonstrated that the age of participating endoscopists is a significant independent predictor for work-related musculoskeletal health issues. Furthermore, the working time in endoscopy and professional experience were related to an increased risk for MSK health disorders. These results correspond with previous systematic reviews of predictors for musculoskeletal health issues in GI endoscopists^8,43^.

The results of the present study show a slightly higher prevalence of work-related musculoskeletal health issues in German endoscopists (76.8%) than in the European survey of R. Morais et al.^19^ with 69.9% affected respondents. By comparison, a nationwide Japanese study showed a prevalence of 43% endoscopists with musculoskeletal health issues^20^, and surveys regarding work-related musculoskeletal health problems in US-American endoscopists showed a prevalence of 47%^6^ to 53%^9^. All nationwide surveys had in common with the present study that the most affected body parts were the shoulder, thumb, neck and back^6,9,19,20^.Compared to other professions, the prevalence of MSK health disorders is considerable higher in GI endoscopists than in other endoscopic disciplines like bronchoscopists (50.6% in a survey among US bronchoscopists performed by Gilbert et al.^44^), but comparable or lower in comparison to dentists^45^ and veterinarians^46^. This might be explained by the difference in the proportion of interventional activity in these professions with comparable risk factors (deficiencies in ergonomic workplace design, overuse injury by repetitive procedures and preservation of awkward body positions during interventions).

Interestingly, our results didn’t show a specific endoscopic procedure to be stronger associated to the occurrence of MSK health disorders despite the differing complexity between, e.g., a diagnostic EGD and an interventional ERCP. As an explanation, it must be considered that the general mechanism of musculoskeletal health impairment is characterised by an overuse injury through repetitive examinations which is equally present in a high volume of repetitive non-complex endoscopic procedures as well as fewer, difficult procedures. However, for further studies it must be admitted that musculoskeletal health disorders and consequently ergonomic implications might differ between distinct types of examination. For example, endoscopists that perform a high volume of short, repetitive EGDs might be more concerned by MSK health problems of the back while endoscopists which predominantly perform ERCP might be more affected by disorders of the thumb and the wrist. This issue has been addressed by procedure-specific ergonomic recommendations by Shergill and colleagues^12^.

It should also be noted that with increasing complexity and interventional opportunities, work-related health hazards, especially regarding musculoskeletal disorders, will increase. In contrast to the implementation of new imaging techniques and artificial intelligence to improve diagnostic and interventional results, ergonomics in GI endoscopy has not been improved to the same extent. Consequently, the further development of preventive approaches for musculoskeletal health disorders will be mandatory to maintain the high volume and quality of endoscopic procedures.

To improve patient and interventionalists safety, several fundamental changes must be implemented. Karsh et al. have proposed to start the prevention of healthcare professionals work-related impairment and the improvement of patients’ safety by redesigning healthcare systems in consideration of hazards which are inherent to the pre-existing system (human factors engineering paradigm)^47^. Consequently, improving structural conditions and endoscopic devices in GI endoscopy will probably lead to a higher safety of patients and interventionalists. To improve ergonomics of endoscopic devices, several pre-existing approaches that address the health hazard of working tools could be transferred to prevent musculoskeletal health issues in GI interventionalists, e.g., from agriculture sector^48^ or from waste collection workers^49^. In addition to ergonomic changes regarding endoscopic devices and structural conditions, our results show that MSK health hazard can be reduced by the implementation of behavioural and administrative interventions: As working time and age of participating endoscopists were associated with a higher risk for MSK health issues, interventionalists might profit from job rotation models (e.g., reduction of endoscopic activity at a certain age) and ergonomic education supported by personal protective equipment (e.g., joint protection).

Despite these results, several limitations of this study must be considered.

First: Hence, GI endoscopy is the major part regarding the volume of endoscopic procedures, the present survey was limited to GI endoscopy specialists, while other endoscopic disciplines, e.g., bronchoscopists, were not included. Furthermore, due to study announcements, members and receivers of the online newsletter and the journal of the German Society for Gastroenterology and the German Society for Endoscopy and Imaging Techniques participated in the present survey. This might exclude endoscopists who were not involved in one of the participating societies to a certain extent. Furthermore, endoscopists that are not affected by work-related health issues might not have participated in the study a priori.

Moreover, it must be stated that an objective verification of the study data was not possible due to the study design as a free-access anonymous online survey (no verification as a practicing GI endoscopist was obtained). Therefore, all answers were checked regarding their plausibility. Nevertheless, it must be admitted that this limitation must be considered regarding the validity of our results.

Third, no control group was recruited to compare the extent of musculoskeletal health problems with other medical or nonmedical disciplines.

In conclusion, the data reveal the high rate of musculoskeletal disorders. Consequently, the further implementation of ergonomic improvement and preventive methods will be necessary to keep pace with the rapidly growing volume and complexity of interventional GI endoscopy. Further interventional studies are mandatory to prevent work-related MSK health disorders in high-volume GI endoscopists.

## Conclusion

The present study has addressed work-related musculoskeletal health disorders in German GI endoscopists for the first time. It was demonstrated that musculoskeletal health issues occur with a high prevalence in German high-volume endoscopists, leading to 82.8% (*n* = 125) participants suffering from musculoskeletal health issues while 76.8% (*n* = 116) participants stated a causal relationship between their endoscopic activity and the reported symptoms. As a result, a strong impact on absence from work (*n* = 15; 9.9%), personal health impairment by the need for medical treatment (*n* = 54; 35.8%) and reduction of leisure time activity (*n* = 55; 36.4%), and work performance by reduced work capacity (*n* = 24; 15.9%) has been demonstrated by our data. The age of participating endoscopists was identified to be significantly associated with an increased risk for the development of MSK health disorders (OR 1.05; 95%-CI 1.00–1.09; *p* = 0.04) while the professional experience (OR 1.04; 95%-CI 1.00–1.09; *p* = 0.06) and working time in endoscopy (OR 1.20; 95%-CI 1.00–1.37; *p* = 0.05) were identified as risk factors with a barely non-significant effect.

As a result of our work, risk groups have been identified (older and/or experienced endoscopists, endoscopists with high working time) who might benefit from specific health interventions, e.g., job rotations models and specific ergonomic interventions more than other practicing endoscopists. In general, the high prevalence of work-related musculoskeletal health issues indicates the necessity for ergonomic workplace optimization in GI endoscopy.

Overall, further interventional and observational studies for the prevention of work-related MSK health issues are widely requested by participating endoscopists and are mandatory to improve personal health and work performance.

## Supplementary Information

Supplementary Information.

## Abbreviations

BMI: Body Mass Index
CI: Confidence Interval
DASH: Disability of the Arm, Shoulder and Hand
DGE-BV: Deutsche Gesellschaft für Endoskopie und bildgebende Verfahren/German Society for Endoscopy and Imaging Methods
DGVS: Deutsche Gesellschaft für Gastroenterologie, Verdauungs- und Stoffwechselkrankheiten/German Society for Gastroenterology
EGD: Oesophagogastroduodenoscopy
EMR: Endoscopic mucosal resection
ESD: Endoscopic submucosal resection
ERCP: Endoscopic retrograde cholangiopancreaticography
EUS: Endoscopic ultrasound
FTR: Full thickness resection
GI: Gastrointestinal
MSK: Musculoskeletal
OR: Odds Ratio
POEM: Peroral endoscopic myotomy
RMDQ: Roland and Morris Disability Questionnaire
SD: Standard Deviation
WOMAC: Western Ontario and McMaster Universities Osteoarthritis Index
