# Supplementary material for: The impact of endoscopic activity on musculoskeletal disorders of high-volume endoscopists in Germany
Source: Sci Rep. 2022 May 20;12:8538. doi: 10.1038/s41598-022-12400-4 (PMC9123012; doi:10.1038/s41598-022-12400-4)
Supplement: Supplementary file 1 — Supplementary Information. [file 41598_2022_12400_MOESM1_ESM.docx]

Table S1: Neck disability index of pain intensity and impairment due to musculoskeletal neck problems of participating endoscopists on a scale from 1=no problems to 6=worst impairment. Neck Disability Score from 0% (no impairment) to 100% (worst impairment)

| **Category of impairment** | **Mean ±SD** | **Range (1-6)** |
| --- | --- | --- |
| Pain intensity (n=78) | 3.0±0.6 | 2-4 |
| Work deterioration (n=78) | 1.5±0.7 | 1-4 |
| Concentration (n=78) | 1.4±0.7 | 1-6 |
| Reading (n=76) | 2.0±0.8 | 1-4 |
| Lifting loads (n=77) | 1.5±0.8 | 1-4 |
| Car driving (n=77) | 1.6±0.7 | 1-4 |
| Leisure time activity (n=77) | 1.8±0.7 | 1-4 |
| Sleep (n=77) | 2.1±0.9 | 1-4 |
| Body care (n=77) | 1.2±0.4 | 1-2 |
| Headache (n=77) | 2.0±0.8 | 1-4 |
|  | **Mean ±SD** | **Range (0-100)** |
| **Neck Disability Index Score (%)** | 34.8±8.1 | 22-62 |

Table S2: Disabilities of the Arm, shoulder and hand (DASH) index for pain intensity and impairment due to upper extremities problems of participating endoscopists on a scale form 1=no problems to 5=worst impairment. Quick-DASH-Score from 0 (no impairment) to 100 (worst impairment).

| **Category of impairment** | **Mean ±SD** | **Range (1-5)** |
| --- | --- | --- |
| Opening an airtight glass jar (n=102) | 1.5±0.8 | 1-4 |
| Heavy housework (n=105) | 1.5±0.7 | 1-4 |
| Carrying a suitcase (n=105) | 1.5±0.7 | 1-4 |
| Washing the back (n=105) | 1.5±0.7 | 1-4 |
| Using a kitchen knife (n=105) | 1.2±0.5 | 1-3 |
| Sportive activities with pressure on shoulder and arms (n=105) | 2.1±1.1 | 1-5 |
| Social activities (n=105) | 1.7±0.6 | 1-4 |
| Work and daily activity (n=105) | 1.6±0.7 | 1-4 |
| Pain intensity (n=105) | 2.2±0.8 | 1-4 |
| Grade of paresthesia (n=104) | 1.5±0.7 | 1-4 |
| Sleep (n=105) | 1.5±0.7 | 1-4 |
|  | **Mean ±SD** | **Range (0-100)** |
| **Quick-DASH-Score** | 19.5±13.4 | 2.5-67.5 |

Table S3: Western Ontario and McMaster Universities Osteoarthritis Index (WOMAC) for pain intensity and impairment due to hip and knee problems of participating endoscopists on a scale form 1=no problems to 10=worst impairment. WOMAC-Score from 0% (no impairment) to 100% (worst impairment).

| **Category of impairment** | **Mean ±SD** | **Range (1-10)** |
| --- | --- | --- |
| **Pain intensity**  Walking on flat ground (n=27)  Climbing stairs (n=27)  In bed at night (n=26)  Sitting/Lying (n=26)  Standing (n=26) | 2.1±1.5  2.9±2.1  2.4±1.6  1.9±0.9  2.2±1.1 | 1-7  1-8  1-7  1-4  1-5 |
| **Joint stiffness**  At morning (n=27)  After resting/sitting/lying (n=27) | 2.3±1.3  2.2±1.1 | 1-5  1-6 |
| **Impairment of daily activities**  Climbing stairs down (n=27)  Climbing stairs up (n=27)  Stand up (n=27)  Standing (n=27)  Bend down (n=27)  Walking on flat ground (n=26)  Get out/in the car (n=27)  Shopping (n=27)  Put on socks (n=27)  Take off socks (n=27)  Get out of bed (n=27)  Lying in bed (n=26)  Get in/out the bathtub (n=26)  Sitting (n=27)  Going to the toilet (n=26)  Heavy activities (n=27)  Easy activities (n=27) | 2.5±1.7  2.1±1.4  2.2±1.2  2.2±1.3  2.3±1.7  1.9±1.3  2.1±1.2  1.5±0.9  1.9±1.4  1.7±1.2  1.7±0.8  1.8±1.1  1.7±1.0  1.6±0.8  1.3±0.7  3.0±1.8  1.7±0.9 | 1-8  1-7  1-6  1-5  1-8  1-6  1-6  1-5  1-6  1-5  1-4  1-6  1-4  1-4  1-4  1-8  1-5 |
|  | **Mean ±SD** | **Range (0-100)** |
| **WOMAC Score (%)** | 19.9±6.6 | 10.4-33.3 |

Table S4: Roland and Morris Disability Questionnaire (RMDQ) for impairment due to back problems of participating endoscopists (n=76). RMDQ-Score from 0 (no impairment) to 24 (worst impairment)

| **Category of impairment** | **Frequency** | **Percentage (%)** |
| --- | --- | --- |
| Body posture  Changes necessary  Sitting position necessary  Lying position necessary | 61  0  0 | 80.3  0.0  0.0 |
| Impairment of activities  Necessary daily activities  Leaving the house  Walking  Standing  Kneeing/Bending down  Turn in bed  Put on/take off socks | 0  2  2  2  16  8  14 | 0.0  2.6  2.6  2.6  21.1  10.5  18.4 |
| Climbing stairs  Support by handrail necessary  Increased time requirement | 3  5 | 3.9  6.6 |
| Increased recovery demand | 5 | 6.6 |
| Stand up  Lying position  Sitting position | 7  2 | 9.2  2.6 |
| Job performance impairment  Support by colleagues necessary  Avoiding heavy work | 2  16 | 2.6  21.1 |
| Cloth changing  Increased time requirement  Need for help | 9  0 | 11.8  0.0 |
| Constant pain | 2 | 2.6 |
| Decreased appetite | 0 | 0.0 |
| Decreased stamina | 0 | 0.0 |
| Sleep impairment | 5 | 6.6 |
| Negative mood | 12 | 15.8 |
|  | **Mean ±SD** | **Range (0-24)** |
| **RMDQ-Score** | 2.6±1.8 | 1-8 |
